# Challenging Notions of Academic Entitlement and Its Rise among Liberal Arts College Students

**DOI:** 10.3390/bs7040081

**Published:** 2017-12-06

**Authors:** Debra Lemke, Jeff Marx, Lauren Dundes

**Affiliations:** 1 Department of Sociology, McDaniel College, Westminster, MD 21157, USA; dlemke@mcdaniel.edu; 2 Department of Physics, McDaniel College, Westminster, MD 21157, USA; jmarx@mcdaniel.edu

**Keywords:** academic entitlement, grading, participation, effort, improvement, mastery, gender, engagement, grit

## Abstract

To assess academic entitlement, we employed a repeated cross-sectional design to compare survey data from two systematic random samples collected eight years apart, in 2009 (*n* = 225) and 2017 (*n* = 159), at a small, private, mid-Atlantic liberal arts college. According to an entitlement scale (based on Greenberger et al., 2008), students were less likely to be entitled in 2017 (27%) than in 2009 (41%) (*p* = 0.02). In 2009, a higher proportion of males than females felt entitled (50% versus 34%, *p* = 0.05), a sex difference that disappeared by 2017. To explore academic entitlement further, we developed the “PIE” scale to measure the extent to which students believe “participation,” “improvement” and “effort” should help determine their course grades. Although the proportion of above average PIE scorers was stable from 2009 (36%) to 2017 (34%), in 2017, more females than males were above average on PIE (26% of males versus 44% of females, *p* = 0.02). PIE, or the desire for recognition of “academic sweat equity,” could reflect students’ support for a learning model that goes beyond mastery and is more developmental and process oriented. These data challenge common conceptions of what constitutes academic entitlement, the belief that it is rising, and suggest continued discussions of what factors should determine grades.

## 1. Introduction

College students are allegedly becoming increasingly self-important [1], narcissistic [2,3] and entitled [4], qualities that tax faculty patience [5,6] and raise questions about the tolerance of such traits among students’ future employers [7]. Excoriation of the “Me Generation” [8,9] includes reports of students pressuring faculty to succumb to relaxed academic standards as well as students’ consumer/customer-oriented mentality [10,11,12,13,14,15,16,17]. Despite contrasting data that show low levels of college student entitlement (e.g., [18]), media outlets have decried the entitled mentality of millennials responsible for “a changed relationship between the schools and the schooled [in] one of the most striking transformations in higher education over the last quarter-century” in which “[s]tudents get the message that they call the shots” ([19], para 5 and 15).

Within the range of problematic behaviors that can potentially comprise academic entitlement, we examined one aspect of the phenomenon that can roughly be described as “expecting an unjustified reward not based on academic achievement” ([20], p. 56). In conducting our assessment of student academic entitlement, we turned to a seminal article on this topic by Greenberger et al. [21] who define academic entitlement as “expectations of high grades for modest effort and demanding attitudes towards teachers” (p. 1193) (a trait they link to narcissism). While two thirds (66%) of their respondents thought that “trying hard” should be considered in a course grade, about one third thought that merely attending most classes should earn them a B in a course.

Greenberger et al.’s [21] results were replicated by Schaefer et al. [22] who also found that the same proportion of students (66%) thought their course grade should reflect their “trying hard,” in a sample of students from a large for-profit online academic institution. Schaefer et al. [22] also reported that a high proportion (80%) thought that if they completed “most of the reading” for a class, they deserved at least a C or better. Eighty-three percent (83%) believed that if they had participated in 70% of more of course activities, they deserved at least a C or higher ([23], p. 83). Over half (55%) agreed that, “On those occasions when my final course grade is lower than I expected, the professor should be willing to allow me to do an additional assignment for a better grade” ([22], p. 83).

Cairns [23], however, notes the complexity of entitlement given the role of the economic climate and job market. He challenges common assumptions about entitlement: “The age of entitlement myth wrongly collapses distinct forms of deservingness into a universally negative category. It is likely that, left unchallenged, the myth has a narrowing effect on political debate” ([23], p. 145).

In this study, we examine the nature and trajectory of academic entitlement, including gender differences given that males are reportedly more likely to be entitled than females [24,25,26] and suffer from a lower retention rate [27]. In considering Cairn’s claim that academic entitlement is wrongly portrayed as a universally negative category, we examine student views of the appropriate role of participation, improvement and effort (PIE) in grading in both 2009 and 2017 and whether the desiderata of PIE even constitute academic entitlement. In contrast to academic entitlement that is clearly undesirable, the role of PIE is potentially positive, especially due to its connection to student engagement, a factor predictive of retention [28]. We used a repeated cross-sectional design to assess how academic entitlement and PIE changed over an eight-year period allowing two consecutive, four-year student cohorts to graduate in order to determine the stability of entitlement and PIE over time.

## 2. Methods

### 2.1. Survey Sample

Our survey data were collected in spring 2009 and spring 2017 at a small, private, mid-Atlantic liberal arts college with about 1350 residential undergraduate students. A total of three hundred and eighty-four (*n* = 384) undergraduate students (52% of whom were female) comprised the two separate systematic random samples that completed an IRB-approved two-page survey in spring 2009 (*n* = 225, 77% response rate) and spring 2017 (*n* = 159, 55% response rate). No changes were made to the 2009 instrument in order to attain measurement invariance and avoid differential item functioning in 2017 (addressed below in Section 2.3: Construct Validity of Scales). Two different samples of respondents were queried, eight years apart, in order to assess historical trends in student conceptions of entitlement over a period in which the two groups of respondents were separated by two four-year student cohorts.

In 2009, student volunteers from a course, Research Methods in Sociology, collected the data while in 2017, student volunteers from the senior seminar course, Capstone: Critical Inquiry, solicited participants (because the methods course was not offered in spring of 2017 due to a sabbatical rotation). Students collecting the data in both years were offered a small amount of extra credit for either their methods or capstone course grades, although most claimed that their willingness to help was more about “survey karma.” The survey consisted of 18 items inspired by Greenberger et al. [21] and additional demographic information (sex, class rank, GPA and major). See Appendix A for the survey instrument.

The entire residential student population was part of the sampling frame that excluded only commuter students (who constituted about 15% of the total undergraduate student population of 1630). Student researchers collecting data were required to return up to three times to a residence hall room assigned to them to solicit participation from one resident in each designated room. After a resident (respondent) agreed to take the survey, that person completed a consent form that was returned to the student researcher. The respondents then filled out the survey, while the student researcher waited a short distance away. The respondents then placed the survey in an envelope that the respondent sealed and then placed inside a larger envelope to assure anonymity.

### 2.2. Operationalization of Mastery, Scales for Entitlement and Participation, Improvement, Effort (PIE)

#### 2.2.1. Operationalizing Mastery

We used the entire dataset, comprised of both 2009 and 2017 data, to recode the importance of mastery in determining a course grade, scored as 0–10, into two levels based on the frequency distribution that revealed a very low number of students who selected seven or lower. As a result, mastery was analyzed as being either below average (0 to 8, 48% of responses) or above average (9–10, 52% of responses: 16% selected 9 and 36% selected 10) (see item 9, part a on our survey in Appendix A).

#### 2.2.2. Academic Entitlement Scale

The same combined dataset was used to devise scales for entitlement based on items 1–5 on our survey (see Appendix A) that were derived from an entitlement scale by Greenberger et al., 2008 [21]. Our five-item academic entitlement scale was based on items from Greenberger et al.’s academic entitlement scale consisting of 15 items (see [21], Table 1, p. 1196). We selected five grade-related items for which at least 25% of Greenberger et al.’s respondents agreed [21]. We excluded two additional items in [21] that met this criterion because we questioned their face and content validity. In other words, these two items were arguably general entitlement or narcissism rather than academic entitlement: (1) I feel I have been poorly treated if a professor cancels an appointment with me on the same day as we were supposed to meet (41% endorsed); (2) Professors who won’t let me take an exam at a different time because of my personal plans (e.g., a vacation or other trip that is important to me) are too strict (30% endorsed).

The scale for entitlement was coded by assigning greater value to the higher letter grades and the more affirmative answers—e.g., for item 1 (see Appendix A), students who answered “probably yes,” that they deserved at least a slightly higher grade if they explained to a professor that they were trying hard in a course, received a numerical value of 3, while a student who responded “probably no” received a value of 2. For items 2 and 3 (about attending and participating—with and without reading, see Appendix A), graded responses were coded similarly with a 5 assigned for an A and a 1 assigned for an F. For items 4 and 5 (about lower than deserved grades on assignments and exams, see Appendix A), coded responses ranged from 5 for “always” to 1 for “never.” Respondents’ entitlement score was the sum of their answers for each of the five questions. The range of summed scores was 5 (the lowest possible entitlement score) to 19 (the highest score coded, with 24 as the highest entitlement score possible). The mean score was 11.5 (with a median of 12). The summed responses were assigned to one of three categories: below average: 10 and below (37%), about average: 11–12 (27%) and above average: 13 and above (36%).

#### 2.2.3. Participation, Improvement, Effort (PIE) Scale

PIE was derived from the role that respondents thought the following three items should play in grading: participation, improvement and effort, scored 0 (not important) to 10 (very important) (see Appendix A, item 9, parts b-d). PIE was calculated by summing individual responses to these three items, with a mean score of 21. These scores were then recoded into the categories of below average: 18 and below (31%), about average: 19–23 (34%) and above average: 24 and above (36%).

### 2.3. Construct Validity of Scales

The construct validity of PIE and Entitlement was established using factor analysis. The Kaiser-Meyer-Olkin measure of sampling adequacy was 0.619—above the recommended value of 0.6—while Bartlett’s test of sphericity was significant (χ^2^ = 690.015, *p* < 0.001). All the communalities were above 0.4 while the average communality was 0.67 (in accordance with a recommended average communality of greater than 0.6 in conjunction with a sample size that exceeds 250 [29].

One of the three factors we identified was comprised of the items we named PIE (see item 9, part b, c, d, Appendix A). The other two factors were components of our Entitlement scale. One of these two factors, which we refer to as engagement, was based on students’ view of the extent to which communicating commitment to a professor, as well as attendance, participation and reading, should determine a grade (see items 1–3 in Appendix A). The other factor comprising the Entitlement scale, that we refer to as perceptions of grading validity, assessed whether students believed that graded products reflected their worth (items 4–5 in Appendix A). These dimensions of our Entitlement scale were consistent with the construction of the concept of academic entitlement established by Greenberger et al. (2008) [21].

## 3. Results

Including survey data from both 2009 (*n* = 225) and 2017 (*n* = 159), the total sample was 384. The smaller number of surveys collected in 2017 reflects a lower rate of students answering their doors but an almost identical rate of student refusals to participate, about 10%, in both years. This low refusal rate is likely linked to a small campus environment. In both samples combined, the overall percent of males was 48% (44% male in 2009 and 54% male in 2017).

### 3.1. Comparison with Data from Greenberger et al. (2008)

Consistent with Greenberger et al. (2008) [21], the exact same percent of our 2009 sample, 66%, agreed that informing a professor about trying hard in a class should slightly boost a grade (item 1 of our survey instrument; see Appendix A). Notably, in our 2017 sample, only 54% agreed, a statistically significant drop (*p* = 0.02) (see Appendix B), challenging the notion of an increasingly entitled undergraduate student population. In [21], 41% of respondents thought they deserved a B for completing most of the reading in a course, compared to 6% (2009) and 10% (2017) in our study (item 2 on our survey instrument, in which we added attendance and participation instead of just asking about a grade deserved for completing most of the reading; see Appendix A). Fewer of our students than Greenberger et al.’s thought they deserved a B just for showing up for a class: only 3% (2009) and 4% (2017), much lower proportions than what Greenberger et al. reported (34%). These low percentages (3% and 4%) were in spite of our modifying Greenberger et al.’s items in our survey to include participation, consistent with norms at a small school with class size of 25 or fewer students (item 3 on our survey instrument; see Appendix A). In the case of students who believed they frequently received lower grades than they deserved on assignments, there was no significant change—9% in 2009 compared to 3% in 2017—much lower than what Greenberger et al. reported (32%) (item 4 on our survey instrument; see Appendix A). We also found significant differences from Greenberger et al. in the proportion of students who said they frequently received lower grades than they deserved on exams, 25% in their study compared to only 7% of our respondents in 2009 and 4% in 2017 (see Appendix A, item 5). Because of the unrealistic scenario in items devised by Greenberger et al. that left open the possibility that extremely minimal effort would actually receive course credit, we modified these items to add participation (see Appendix A, items 4 and 5). Therefore, given this addition (of participation), we might have expected more students, not fewer than in Greenberger et al. to think that they deserved a B. Instead, only a small fraction of our sample believed that such modest efforts merited a B, in either 2009 or 2017.

### 3.2. Academic Entitlement Scale

Like most of our data from individual questions cited above, our entitlement scale revealed that respondents in 2009 were more much more likely to be entitled than in 2017 (see Table 1).

In accordance with [21], we found only minor associations between demographic variables and academic entitlement, with the exception of sex. Sex (but not year in school or GPA) predicted entitlement as operationalized by our scale: males were significantly more likely than females to be entitled in 2009 (50% versus 34%; χ^2^ = 5.95, *p* = 0.05) but not in 2017 (28% versus 27%).

Women were also much more likely than men to prioritize academics over social life or sports. (We also included extra-curricular activities as a “priority” but only 5% of the total sample selected this option; see Appendix A, item 18). This sex difference was especially marked in 2009, when sex alone accounted for 13% of the variance in the priority selected: 41% of males versus 71% of females prioritized academics (χ^2^ = 28.41, *p* < 0.001). This gender gap was still statistically significant but much narrower by 2017 (70% of males versus 87% of females prioritized academics, *p* = 0.02). This finding helps validate the operationalization of our entitlement scale that identified males as more entitled than women in 2009 but not in 2017.

The finding that only 41% of males prioritized their education over their social life and sports in 2009 suggests that men valued their education less than women at a time when men were also more entitled. As a corollary, males were also more likely to say graduating was more important than learning in 2009 (58% of males versus 45% of females, χ^2^ = 3.68, *p* = 0.05) but not in 2017 (52% of males versus 51% of females) (see Appendix A, item 13).

### 3.3. Participation, Improvement and Effort Scale (PIE)

Our creation of a second scale to explore the complexity of academic entitlement, that we call PIE, helped explain why our findings countered reports and perceptions of increased college student entitlement. In this second scale measuring PIE (see Appendix A, item 9), effort was the most central concept, given the largest drop in the Cronbach’s alpha if it were deleted (see Table 2).

PIE, which we also refer to as “academic sweat equity,” changed very little from 2009 to 2017. In particular, Table 3 reveals that the proportion of respondents that were above average on the PIE scale in 2009 and 2017 was almost unchanged (36% and 34% respectively). We also examined whether sex differences emerged in our PIE scale. Although the differences by sex were not statistically significant in the above average PIE group in 2009 (32% of males and 40% of females), a statistically significant difference by sex did emerge in 2017: 26% of males and 44% of females were above average on the PIE scale (χ^2^ = 7.50; *p* = 0.024). Thus, the difference by sex in those who were above average on the PIE scale appeared only in 2017 (denoting that women were much more likely than men to believe “academic sweat equity” should be reflected in their grades).

Our analysis did not reveal any significant differences in either our entitlement or PIE scales by GPA, a finding consistent with results from both Greenberger et al. [21] as well as Bonaccio et al. [30], whose research indicates that academic entitlement does not predict course grades once personality and general mental ability are taken into account.

### 3.4. The Importance of Good Grades to Signal Understanding of Course Material

Besides sex, another variable correlated with above average scores on the PIE scale: agreement that “good grades are important to you because they give you at least one indicator that you understand the material” (answered on a four-point scale from strongly agree to strongly disagree; see Appendix A item 14, part d). This variable, which we refer to as, “Signals Understanding,” was strongly predictive of PIE (see Table 4).

Almost half (46%) of those who strongly agreed that good grades are important because they signal understanding of course material also scored above average on the PIE scale (33% of males and 51% of females, a non-statistically significant difference among this subgroup (*p* = 0.07). Therefore, PIE is associated with the desire to get good grades as a source of validation.

The “Signals Understanding” variable, however, does not correlate with mastery (see Appendix B, item 9, part a), suggesting that students who want validation of their understanding of course material see grades as a barometer of their command of the material but not necessarily as an end in and of themselves. Students above average on PIE may not feel “entitled” to good grades as traditionally defined but rather believe that good grades confirm their comprehension. The perception of “good grades” is subject to changes resulting from grade inflation that has been observed in the mid-1990s to the mid-2000s [31]. However, at the institution where data were collected, the average student GPA for all students remained stable: Spring term 2009: 3.094; Spring term 2013: 2.990; Spring term 2017: 2.998.

### 3.5. Above Average PIE Not Linked to Grade Entitlement by Explaining Efforts or a Grading Curve

In addition to students above average on the PIE scale seeking good grades as validation of their understanding of course material (“Signals Understanding”), they are also no more likely to believe that explaining to a professor that they are trying hard in a course should result in getting at least a slightly higher grade: 50% of those who are below average PIE and 50% of those who are above average PIE endorse this means of boosting their grade. Furthermore, there is no statistically significant difference between by PIE in rejecting any kind of curve for exam grades, even when the class average is an F (see Appendix A, item 7, part 6).

### 3.6. Above Average PIE Linked to Above Average Valuing of Mastery

We also found that PIE correlates with mastery, such that students assigning mastery less importance were also more likely to be below average on PIE while students placing a higher importance on mastery (above average, scored as 9–10) were more likely to score above average on the PIE scale (see Table 5). In other words, this latter group expects their grade to reflect both their command of the material *and* their academic sweat equity. The statistically significant link between PIE and mastery suggests that students that are above average on PIE tend to want their efforts to count as a matter of course but *not* in a way that necessarily substitutes for mastery.

As a corollary, Table 5 also reveals that 57% of those who believe mastery is of below average importance also think that PIE should not be important in determining grades. This group could be the most entitled of all; they may feel that graded work is not capable of reflecting their aptitude nor is academic sweat equity necessary given their superiority (a type of narcissism). The attitudes of this same group could be explained in another way as well: they could feel disaffected in college and therefore less willing to invest in the educational process. It could be that students who fall into this category and *not* those who want to be rewarded for PIE, should be targeted for intervention.

### 3.7. Extra Credit Work to Boost Grades and Gender Differences in PIE

To understand why women are more likely to have above average PIE scores but only in 2017, we examined whether students believed that outside of exceptional circumstances like excused illness, they should be allowed to do additional relevant work (“extra credit”) to improve their course grade, an item drawn from Greenberger et al. [21] (see Appendix A, item 11). While there was no statistically significant difference between males and females in 2009 in those who answered “absolutely yes” when asked whether they should be able to do additional work to improve their grade (38% of males and 27% of females (*p* = 0.08), in 2017, about half as many males (15%) as females (31%) answered “absolutely yes” to this question (χ^2^ = 6.22, *p* = 0.04). This relationship between PIE and the importance to women of allowing extra credit bolsters support for PIE as tied to the valuation of the learning process. The willingness of women to expend extra energy is further evidence of how academic sweat equity is distinct from entitlement.

## 4. Discussion

### 4.1. Lower Academic Entitlement and the Closing of the Gender Gap in Academic Entitlement

We did not find evidence that academic entitlement is increasing. In fact, our evidence suggests entitlement as traditionally defined has generally decreased from 2009 to 2017, a difference largely driven by the drop in the proportion of males who felt academically entitled in 2009 (50%) compared to 2017 (34%), a much larger change than for females (from 34% in 2009 to 27% in 2017).

Although a gender gap in academic entitlement has been noted in the literature, this gap remains poorly understood [2,21,25,26], prompting calls for more research to determine why males are more entitled, including studies of the role of parental socialization [32]). Our findings that the gap appears to have closed could be partly explained by changing assumptions about the prognosis for success after college. Having a college degree in the job market, while advantageous, does not confer the same advantages as in 2009 when the first sample was surveyed. According to the US Census Bureau and the US Bureau of Labor Statistics, the job market has changed dramatically in terms of the proportion of job postings for college and non-college jobs; in April 2009, the number of postings was almost the same for college and non-college jobs, a contrast with the most recent data available (December 2015) that reveal 1.34 times more non-college jobs than college jobs that were posted [33]. These data parallel a rise in the percentage of college graduates that were unemployed or “underemployed”—working in a job that typically does not require a bachelor’s degree [34]. The less-rosy job prognosis after college could help explain the decline in males’ sense of entitlement, diminishing their notion of “the world is my oyster” related to males’ historical advantage in the job market [35].

### 4.2. Why College Women Are Higher in PIE in 2017

The widening of the PIE gender difference in 2017 (from no gap in 2009 to an 18%-point gap in those who were above average on the PIE scale) could be linked to the change in the campus gender balance in which not only female students but also female faculty and administrators constituted a greater presence in 2017 compared to 2009. Between 1993 and 2013, the rate of full-time faculty appointments of women was five times that of men, resulting in the near elimination of the gender gap among tenure-track faculty at most institution types [36], including the institution examined in this study.

Within an environment of increasing female authority figures that incorporates women’s more collaborative and consensus-building leadership style [37], a higher proportion of female students might expect academic sweat equity to count in grading, consistent with the pedagogical value of emphasizing the learning process [38]. This gender difference could reflect women’s tendency to be more aware of interpersonal connections, feelings and cues [39] especially within a less androcentric environment. In other words, the gender gap on the PIE scale in 2017 may signal that women felt more empowered to seek recognition for their academic sweat equity given its role in the learning process.

This gender difference also could stem from boys’ expectation of praise for outcome performances that demonstrate their abilities (sometimes in the form of winning). In contrast, girls tend to be conditioned to expect kudos for the quality of their performance, that is, process goals or developmental steps that are conveyed in such feedback as, “at least you tried hard” [40]. Eleanor Maccoby [41,42] observed the emergence of these patterns within the context of single-sex groups, in which girls value communication with adults, while boys seek autonomy from adults. This difference was manifested in boys’ emphasis on competence in competitive domains compared to girls’ interest in process, including communication and maintaining harmonious relationships, over outcome.

Gender differences in process or experiential learning may also help explain the wide disparity in the lower percent of males who study abroad (who commonly represent 35% or fewer of participants) [43]. The principal benefit of studying abroad occurs through the experience of living in a different culture. In a highly competitive study abroad program, the underlying philosophy is rooted in a primary tenet of Kolb’s experiential learning theory [44]: “Learning is best conceived as a process, not in terms of outcomes” (p. 108), such that experiential learning transforms students’ experiences into new knowledge and personal growth [43]. The role of experience in learning dovetails with women’s greater interest in interpersonal facets of work such as social contacts and working conditions compared to men’s task orientation [45]. This female-linked orientation seen in both the workplace and possibly the overrepresentation of women in study abroad programs may be associated with the elements of PIE, in which tenacity and involvement are valued, separate from (but not instead of) mastery.

### 4.3. PIE and Retention

Students’ (especially female students’) wish for participation, improvement and effort to count in grading (PIE) could be sustained if educational institutions continue to tout the importance of what we call PIE as part of the prescription for retention, e.g., the value of academic conscientiousness [46,47] and expectations that students spend three hours per credit in outside-of-the-classroom work (as promulgated at the institution studied). While definitions of effort may vary, complicating its accurate measurement, the point is that part of what many academics see as entitlement may in fact be the reasonable expectation of a payoff when students follow the prescription promoted as key to success. In addition, at around the time the first set of data was collected in 2009, the National Survey of Student Engagement (NSSE) [48] (with 2017 data from 725 colleges and universities) proposed that because students “learn more when they are intensely involved in their education—inside and outside the classroom, [faculty] are going beyond the student-as-sponge model in designing courses” ([49], para 15). This movement, underway in 2009, may have helped spawn the emphasis on the elements of PIE (stable from 2009 to 2017) that are promoted to increase student engagement in an effort to attract and retain college students. Measures of engagement from NSSE paralleled our 2017 gender difference in PIE in that women in NSSE were more likely to review their notes after class and to be challenged by courses to “do their best work” (see NSSE [48], 2017, items 9b and 10).

### 4.4. Is PIE Entitlement?

Among the third of students from both 2009 and 2017 who were above average on PIE, there is a corresponding tendency for them to see good grades as a signal of their understanding of course material. Yet are these students nevertheless entitled? In answering this question, we should keep in mind that students who want academic sweat equity to count in grading are not those more likely to have low GPAs, nor to downplay the importance of mastery in grading. In other words, students above average on PIE do not tend to expect PIE to substitute for mastery in earning grades.

### 4.5. PIE as Non-Exploitative Entitlement

These results are consistent with the findings of Lessard et al. [13] who parse the psychological concepts underlying entitlement to reveal that higher levels of what the authors call “exploitative entitlement” relate to the belief that students should be able to put in less effort for the same rewards as others. In contrast and of relevance to our findings, non-exploitive entitlement describes students that feel that they have a “legitimate right to positive outcomes” (akin to our above average PIE students who want their effort to count in grading) (p. 529). Not only did Lessard et al. [13] find that non-exploitive entitlement does *not* correlate with the expectation for special treatment but they also discovered that non-exploitive entitlement is associated with prosocial attributes. They conclude that because of the unrecognized complexity of the concept of entitlement, the negative portrayal “anecdotally attributed” to all dimensions of entitlement “may be off the mark” ([13], p. 529). We believe that students scoring above average on the PIE scale may also be prosocial while their desire to get credit for the process could be mislabeled entitlement.

### 4.6. Value of PIE Given Limitations of Grading Mastery

Since students who are above average on the PIE scale do still care about mastery, PIE could be less reflective of entitlement and more of a reaction to the vagaries of grading. In other words, PIE could help mitigate the uncertainties inherent in grading mastery by providing an additional means of academic validation. Demonstrating mastery is subject to a host of factors that affect student performance on exams and assignments (e.g., student illness, anxiety, distraction) as well as instructors’ sometimes-idiosyncratic grading. With the focus on conducting research in graduate school, pedagogy, including grading, may sometimes be relegated to a secondary role at best.

Even in courses in which a professor is internally consistent in grading, inter-professor variation is inevitable in a system where professors have discretion in how they run their classes. The notion that assessment measures should rely on mastery over process is reminiscent of Freire’s [50] critique of an educational system in which instructors deposit knowledge into passive students who are oppressed by instructors’ authoritarian rule (see the “student–as-sponge” model referenced above). Furthermore, there is evidence that students who “worked harder” ([51] p. 305), a trait called perseverance of effort, a type of “grit” involving sustained time and energy necessary for accomplishing long-term tasks, predicted all aspects of self-regulated learning [51] and academic achievement [52]. There is some support for the high value of dedication in the contemporary job market. In a survey conducted by a recruitment service company, only 1% of 1427 professionals and executives mentioned GPA as the top attribute sought when hiring a new college graduate, far fewer than the 61% who selected drive and passion as paramount [53].

By including PIE in grading, students can at least get some credit for actions that may not lead to full mastery but nevertheless add to a sense of control that can compensate for professors who do not validate students’ understanding in the grades assigned for mastery. We also should not ignore animal-based research showing that violating expectations, or disrupting the sense of cause and effect (or agency), can lead to frustration [54], parallel to the link between academic self-efficacy and the degree of effort expended [55].

### 4.7. Advantages of PIE as a Significant Part of a Student’s Grade

Instructors who take PIE into account in grading could be seen as acknowledging (1) the importance of the process/journey; (2) how assessment of student knowledge and understanding is imperfect (that is, that measuring mastery is difficult); (3) that ongoing input encompassed by PIE provides more data points for assessment beyond just assignments and exams. The fact that many professors take participation into account in a course grade shows that counting PIE is not anomalous.

In contrast to conventional grades, PIE grants students more control. In feeling more empowered, their sense of agency could grow. Female students in particular may be responding to an increased emphasis on individualized experiences encouraging engagement with course material, consistent with research connecting conscientiousness and work drive with retention among women [56]. We cannot be sure, however, if above average PIE scores reflect students’ desire for a more holistic approach to grading rather than the traditional approach to grading. Nevertheless, the concept of academic entitlement must go beyond dismissive popular conceptions of college students as narcissistic.

### 4.8. Moving Forward

While PIE is not necessarily entitlement, other student beliefs that do constitute academic entitlement merit redress. Modern childhood socialization tends to emphasize a more developmental model, in which students are trained to expect validation: everyone gets a prize for participating [57]; this philosophy could be driving both PIE and academic entitlement. Whether academics like it or not, we cannot turn back the clock and say that only winners should be recognized once students are socialized to think that participation merits a reward. Despite some educators’ resistance to the “everyone gets a trophy” mentality, rewarding involvement nevertheless nurtures grit—and serves as an incentive for increased effort and engagement. Furthermore, we can capitalize on this socialization by teaching productive participation and engagement as a gateway to mastery that also has potential to enhance student retention [58,59].

## 5. Limitations

Results are limited by having only two temporal data points, leaving open the details of how the pattern may have changed over the course of eight years in ways other than a linear shift. In addition, the external validity is related to characteristics of a small liberal arts college with small class sizes (of about 20) that affect the extent to which students are able to participate or communicate to their instructors the effort expended. Furthermore, the school’s small size (of about 1630 residential and commuter students) contributes to a campus atmosphere that undoubtedly creates different expectations for what types of performance should be considered in determining a student’s grade. As a result, these findings may be less applicable to schools with larger class sizes and greater overall student enrollments. To assess the generalizability of these findings, we recommend future studies employ a mixed-methods approach in which qualitative interview data provide context for quantitative survey results.

The lower response rate in 2017 is also worthy of mention. The drop from 77% to 55% could be due in part to survey fatigue, as compared to 2009, more students and faculty conduct on-campus surveys (including Qualtrics surveys and other surveys administered online). Despite the drop in the response rate, however and as mentioned in the results section, student volunteers who collected data in both in 2009 and 2017 reported that of their non-respondents, about 90% were a result of no one answering the door to the dormitory rooms they were assigned in the systematic random sample.

We have no way of knowing, however, whether students were not in their dormitory rooms or did not respond to the knock on their door. Some students may not answer their doors because they may be increasingly likely to assume that anyone who wishes to reach them will text or otherwise alert them in advance [60]. In addition, in an era when millennials may cultivate an image on social media platforms such as Facebook, Snapchat and Instagram [61], they may not be ready to present their public self if someone comes to the door unannounced. In any event, new norms of communication raise the possibility that the lower response rate was a result of the inability to directly speak with students to solicit participation much more so than refusals to participate.

## 6. Conclusions

Perceptions that academic entitlement is a growing problem may not reflect reality or the multifaceted nature of this phenomenon. Recommendations about the optimal balance between mastery and PIE require more data about how students view the grading process and optimal ways in which faculty can allow students to demonstrate how they have benefited from a course. While mastery is clearly important, its measurement is an imperfect science at best. Furthermore, both mastery and PIE predict student retention.

In consideration of PIE and its place in student assessment, we must recognize how to best cultivate meaningful student engagement that can operate synergistically with mastery on the job. Academic sweat equity in the form of hard work and perseverance can sustain students entering the workforce as they attain new skills that relate to the process of achievement that complements mastery. The importance of this process appears to be more salient for women who are more apt to recognize the value in the journey and academic sweat equity than men who may be more outcome-driven than process-driven.

Among both male and female students, there is some expectation that participation, improvement and effort be part of their course grade (PIE), reflecting cultural lore that hard work leads to success (or mastery). If students buy into the belief that academic sweat equity or PIE will result in command of course material, they may seem entitled to professors if PIE is excluded from course grades. Faculty should recognize that they teach more than content. They also teach life skills. Life outside of college is less about whether students have learned how to excel on tests and graded assignments of their particular instructors. Learning is a developmental and lifelong process; when students leave college, they need to be able to return to the control and agency afforded by PIE.

Although punctuated by knowledge milestones, learning does not end at an outcome, nor is it always evidenced in performance. Rather, learning occurs through the course of connected experiences in which knowledge is modified and re-formed. Learning is not just the result of cognition but involves the integrated functioning of the total person—thinking, feeling, perceiving and behaving.

([62], p. 138)

## Figures and Tables

**Table 1:** Entitlement Scale Distribution: 2009 versus 2017.

Entitlement Scale	2009	2017
below average	32%	45%
about average	27%	28%
above average	41%	27%
χ^2^: 8.30, *p* = 0.021	100%	100%

**Table 2:** PIE scale: (Cronbach’s alpha 0.71).

Item	Cronbach’s Alpha If Item Deleted
Participation	0.692
Improvement	0.628
Effort	0.544

**Table 3:** Changes in PIE from 2009 to 2017.

Pie Value	2009	2017
Below average PIE	29%	34%
About average PIE	35%	32%
Above average PIE	36%	34%
total	100%	100%

**Table 4:** PIE & Agreement that good grades are important because they provide at least one sign of understanding course material.

PIE	Signals Understanding		
	Strongly Agree	Agree	Disagree & Strongly Disagree
below average	21%	31%	50%
about average	33%	36%	27%
above average	46%	34%	23%
(χ^2^ = 14.626 *p* = 0.006)	100%	100%	100%

**Table 5:** Importance that Mastery & Participation, Improvement and Effort (PIE scale) should have in course grades.

PIE Importance:	Mastery: Below Average Importance (48% of Total Sample)	Mastery: Above Average Importance (52% of Total Sample)
below average & about average	57%	43%
above average	43%	57%
(χ^2^ = 7.34; *p* = 0.007)	100%	100%

## Appendix A

1.	If you explain to a professor that you are trying hard in a course, then you deserve at least a slightly higher grade:				
	Absolutely yes	Probably yes	Probably not		Absolutely not
2.	If all you do in a course is attend and participate in every class session **and** complete most of the reading for the class but do **not** complete the assignments or pass any exams, then you deserve a minimum grade in that course of a …				
	A	B	C	D	F
3.	If all you do in a course is attend and participate in every class session but you do **not do the reading**, complete the assignments, nor pass any exams, then you deserve a minimum grade in that course of a …				
	A	B	C	D	F
4.	Professors give you significantly lower grades than you deserve on individual assignments (excluding exams) …				
	never	rarely	occasionally	frequently	always
5.	Professors give you significantly lower grades than you deserve on exams…				
	never	rarely	occasionally	frequently	always
6.	If you need a certain GPA to maintain a scholarship, etc., should a professor take that into account when determining your final course grade?				
	Absolutely yes	Probably yes	Probably not		Absolutely not

7.		Generally, you think that a professor should grade on a curve when the class average on an exam is at or below a …						
		A	B	C	D	F	Generally, grades should not be curved	
8.	a.	For a typical class that meets 3 h/week, about how many additional hours per week outside of class (at most) should you need to spend on coursework…						
			~0–1 h.	~2–3 h.	~4–5 h.	~6–9 h	~10–12 h	12+ h
		… to get an A						
		… to get a C						
		… to get a D−						
	b.	Thinking about a typical student (not specifically about you), most of your professors would respond:						
			~0–1 h.	~2–3 h.	~4–5 h.	~6–9 h	~10–12 h	12+ h
		… to get an A						
		… to get a C						
		… to get a D−						

9.		How important do you think the following should be when a professor determines your course grade?											
			0	1	2	3	4	5	6	7	8	9	10
	A	Your mastery of the material as shown through graded work: tests, assignments, papers, labs, etc.											
	B	Your participation in class											
	C	Your effort spent outside of class											
	D	Your improvement in the subject											

10.		Barring exceptional circumstances (such as an excused illness), if you asked a professor from a previous semester if you could do additional, relevant work (“extra credit”) to improve your course grade, then he/she should allow you to do so:			
		Absolutely yes	Probably yes	Probably not	Absolutely not
11.		Barring exceptional circumstances (such as an excused illness), while enrolled in a class, if you asked the professor if you could do additional, relevant work (“extra credit”) to improve your course grade, then he/she should allow you to do so:			
		Absolutely yes	Probably yes	Probably not	Absolutely not
12.	a	What do you consider to be good grades?			
		D’s or higher	C’s or higher	B’s or higher	An A is the only good grade
	b	Thinking about a typical student (not specifically about you), most of your professors would respond:			
		D’s or higher	C’s or higher	B’s or higher	An A is the only good grade
13.		If you had to pick one of the choices, which is more important to you?			

14		**Good grades are important to you because…**			Strongly Agree	Agree	Disagree	Strongly Disagree
		…you need them to advance in life (like graduate school or a job).						
		…your family pressures you to achieve them.						
		…you want to make your family proud.						
		… they give you at least one indicator that you understand the material.						
		…you want to fit in or keep up with friends.						
15		Do you worry about your grades?						
		Very much		Somewhat	Not much		Not at all	
16		My family’s budget is…						
		tight	comfortable	very comfortable				
17		What percentage of the overall total cost for your education will you (not other members of your family) be responsible for paying? (Don’t forget about loans that you will need to pay later!)						
		none		up to a third	about half		~2/3	all/almost all
18		Which of the following is most important to you at college? (Choose only one)						
		Academics		Social life	Sports		(Non-sport) extra-curricular activity	
								

Sex		My overall GPA	What is your major or intended major?
M	F		
		0.0–0.99	
Class rank		1.0–1.99	
Senior		2.0–2.49	
Junior		2.5–2.99	
Sophomore		3.0–3.49	
First-year		3.5–3.99	
		4.0 or higher	

## Appendix B

Table of Major Results: 2009 vs. 2017.

**Value of Effort**	**2009**	**2017**
If trying hard, deserve at least a slightly higher grade	66%	54%, *p* = 0.02
**Relevant “extra credit” work should be allowed to improve a grade**		
From a PREVIOUS semester	54%	38%, *p* = 0.002
From a CURRENT semester	87%	76%, *p* = 0.004
**Receive significantly lower grades than deserved on**		
**assignments** occasionally (40%) or frequently (9%) ’09	49%	41% NS
**assignments** occasionally (38%) or frequently (3%) ’17		
**exams** occasionally (34%) or frequently (7%) ’09	41%	29%, *p* = 0.02
**exams** occasionally (25%) or frequently (4%) ’17		
**Professor grading (if student does no assignments, passes no exams)**		
Deserve at least a B (6%) or C (44%) for attending, participating & reading ’09	50%	40%, *p* = 0.05
Deserve at least a B (10%) or C (30%) for attending, participating & reading ’17		
Deserve at least a B (3%) or C (16%) for attending & participating ’09	19%	18%, NS
Deserve at least a B (4%) or C (14%) for attending & participating ’17		
Deserves an F if only attends, participates & reads	10%	15%, NS
Deserves an F if only attends, participates (no reading)	36%	52%, *p* = 0.002
Profs should curve when average is at or below B (8%) or C (40%) ’09	48%	51%, NS
Profs should curve when average is at or below B (7%) or C (44%) ’17		
Profs should take into account a student’s GPA needs for scholarships, ROTC, etc.	41%	41%, NS
**Believes only 0-3 additional outside of class hours needed for a/an**		
A	20%	12%, *p* = 0.04
C	71%	58%, *p* = 0.008
D−	97%	96%, NS
**Believes professors think only 0–3 extra hours needed for a/an**		
A	8%	6% NS
C	38%	35% NS
D−	90%	84% NS
**Believes 6+ additional outside of class hours to get a/an**		
A	42%	53%, *p* = 0.03
C	7%	6%, NS
D−	1%	3%, NS
**Believes professors think 6+ extra hours needed for a/an**		
A	68%	78%, NS
C	23%	30%, NS
D−	4%	6%, NS
**Ranks the following as very important in determining a course grade (ranked 9–10 on 10-point scale):**		
Mastery (9–10: above average)	50%	56%, NS
Participation (ranked 9–10)	29%	25%, NS
Improvement (ranked 9–10)	43%	38%, NS
Effort (ranked 9–10)	27%	29%, NS
Bs are good grades	74%	79%, NS
Believes professors consider Bs to be a good grade	65%	69%, NS
Cs are good grades	12%	13%, NS
Believes professors consider Cs to be a good grade	25%	23%, NS
**Priorities**		
academics	58%	77%, *p* = 0.0001
social life	20%	16%, NS
sports	14%	6%, *p* = 0.01
extra-curricular	8%	1%, *p* = 0.003
Between learning and **graduating**, graduating is more important	51%	52%, NS
**Stress**		
Students who worry very much (46%) or somewhat (44%) about grades ’09	90%	89%, NS
Students who worry very much (54%) or somewhat (35%) about grades ’17		
**Paying for school**		
Students responsible for paying **none** of their total cost for education	30%	20%, *p* = 0.03
Students responsible for paying **almost/all** of their total cost for education	14%	21%, NS
**Strongly agree good grades are important:**		
to advance in life	53%	50%, NS
to make their family proud	43%	42%, NS
to show they understand the material	30%	26%, NS
due to family pressures	20%	26%, NS
to keep up or fit in with friends	10%	10%, NS

## References

[1] Twenge J.M., Carter N.T., Campbell W.K. (2017). Age, time period, and birth cohort differences in self-esteem: Reexamining a cohort-sequential longitudinal study. J. Personal. Soc. Psychol..

[2] Chowning K., Campbell N.J. (2009). Development and validation of a measure of academic entitlement: Individual differences in students’ externalized responsibility and entitled expectations. J. Educ. Psychol..

[3] Westerman J.W., Bergman J.Z., Bergman S.M., Daly J.P. (2012). Are universities creating millennial narcissistic employees? An empirical examination of narcissism in business students and its implications. J. Manag. Educ..

[4] Elias R.Z. (2017). Academic entitlement and its relationship with perception of cheating ethics. J. Educ. Bus..

[5] Jiang L., Tripp T.M., Hong P.Y. (2017). College instruction is not so stress free after all: A qualitative and quantitative study of academic entitlement, uncivil behaviors, and instructor strain and burnout. Stress Health.

[6] Lippmann S., Bulanda R.E., Wagenaar T.C. (2009). Student entitlement: Issues and strategies for confronting entitlement in the classroom and beyond. Coll. Teach..

[7] Peirone A., Maticka-Tyndale E. (2017). “I bought my degree, now I want my job!” Is academic entitlement related to prospective workplace entitlement?. Innov. High. Educ..

[8] Twenge J.M. (2013). Teaching Generation Me. Teach. Psychol..

[9] Twenge J.M. (2014). Generation Me—Revised and Updated: Why Today's Young Americans Are More Confident, Assertive, Entitled-And More Miserable than Ever Before.

[10] Anderson D., Halberstadt J., Aitken R. (2013). Entitlement attitudes predict students’ poor performance in challenging academic conditions. Int. J. High. Educ..

[11] Fullerton D.S., Knowlton D.S., Hagopian K.J. (2013). What Students Say About Their Own Sense of Entitlement. From Entitlement to Engagement: Affirming Millennial Students' Egos in the Higher Education Classroom.

[12] Kopp J.P., Zinn T.E., Finney S.J., Jurich D.P. (2011). The development and evaluation of the academic entitlement questionnaire. Meas. Eval. Couns. Dev..

[13] Lessard J., Greenberger E., Chen C., Farruggia S. (2011). Are youths’ feelings of entitlement always “bad”? Evidence for a distinction between exploitive and non-exploitive dimensions of entitlement. J. Adolesc..

[14] McLellan C.K., Jackson D.L. (2017). Personality, self-regulated learning, and academic entitlement. Soc. Psychol. Educ..

[15] Miller B.K. (2013). Measurement of academic entitlement. Psychol. Rep..

[16] Singleton-Jackson J.A., Jackson D.L., Reinhardt J. (2010). Students as consumers of knowledge: Are they buying what we’re selling?. Innov. High. Educ..

[17] Singleton-Jackson J.A., Jackson D.L., Reinhardt J. (2011). Academic entitlement: Exploring definitions and dimensions of entitled students. Int. J. Interdiscip. Soc. Sci..

[18] Sessoms J., Finney S.J., Kopp J.P. (2016). Does the measurement or magnitude of academic entitlement change over time?. Meas. Eval. Couns. Dev..

[19] Bruni F. (2016). In college turmoil, signs of a changed relationship with students. New York Times.

[20] Jackson D.L., Singleton-Jackson J.A., Frey M.P. (2011). Report of a measure of academic entitlement. Am. Int. J. Contemp. Res..

[21] Greenberger E., Lessard J., Chen C., Farruggia S.P. (2008). Self-entitled college students: Contributions of personality, parenting, and motivational factors. J. Youth Adolesc..

[22] Schaefer T., Barta M., Whitley W., Stogsdill M. (2013). The “You Owe Me!” Mentality: A student entitlement perception paradox. Learn. High. Educ..

[23] Cairns J. (2017). The Myth of the Age of Entitlement: Millennials, Austerity, and Hope.

[24] Boswell S.S. (2012). “I deserve success”: Academic entitlement attitudes and their relationships with course self-efficacy, social networking, and demographic variables. Soc. Psychol. Educ..

[25] Ciani K.D., Summers J.J., Easter M.A. (2008). Gender differences in academic entitlement among college students. J. Genet. Psychol..

[26] Turnipseed D.L., Cohen S.R. (2015). Academic entitlement and socially aversive personalities: Does the Dark Triad predict academic entitlement?. Personal. Individ. Differ..

[27] Ewert S. (2010). Male and female pathways through four-year colleges: Disruption and sex stratification in higher education. Am. Educ. Res. J..

[28] Flynn D. (2014). Baccalaureate attainment of college students at 4-year institutions as a function of student engagement behaviors: Social and academic student engagement behaviors matter. Res. High. Educ..

[29] Kaiser H.F. (1974). An index of factorial simplicity. Psychometrika.

[30] Bonaccio S., Reeve C.L., Lyerly J. (2016). Academic entitlement: Its personality and general mental ability correlates, and academic consequences. Personal. Individ. Differ..

[31] Kostal J.W., Kuncel N.R., Sackett PR. (2016). Grade inflation marches on: Grade increases from the 1990s to 2000s. Educ. Meas. Issues Pract..

[32] Sohr-Preston S., Boswell S.S. (2015). Predicting academic entitlement in undergraduates. Int. J. Teach. Learn. High. Educ..

[33] Federal Reserve Bank of New York (2017). The Labor Market for Recent College Graduates. https://www.newyorkfed.org/research/college-labor-market/college-labor-market_labor-demand.html.

[34] Abel J.R., Deitz R., Su Y. (2014). Are recent college graduates finding good jobs?. Curr. Issues Econ. Financ..

[35] Hogue M., Yoder J.D., Singleton S.B. (2007). The gender wage gap: An explanation of men’s elevated wage entitlement. Sex Roles.

[36] Flaherty C. (2016). More Faculty Diversity, not on Tenure Track. https://www.insidehighered.com/news/2016/08/22/study-finds-gains-faculty-diversity-not-tenure-track.

[37] Herrera R., Duncan P.A., Green M.T., Skaggs S.L. (2012). The effect of gender on leadership and culture. Glob. Bus. Organ. Excell..

[38] Conklin T.A. (2013). Making It Personal: The importance of student experience in creating autonomy-supportive classrooms for millennial learners. J. Manag. Educ..

[39] Kendall S., Tannen D., Tannen D., Hamilton H., Schiffrin D. (2015). Discourse and Gender. The Handbook of Discourse Analysis.

[40] Hyde J.S., Durik A.M., Elliot A.J., Dweck C.S. (2005). Chapter 21: Gender, Competence, and Motivation. Handbook of Competence and Motivation.

[41] Maccoby E.E. (1990). Gender and relationships: A developmental account. Am. Psychol..

[42] Maccoby E.E. (1998). The Two Sexes: Growing up Apart, Coming Together.

[43] Brandauer S.C., Hovmand S. (2013). Preparing business students for the global workplace through study abroad: A case study of the Danish Institute for study abroad. J. Int. Educ. Bus..

[44] Kolb D.A. (1984). Experiential Learning: Experience as a Source of Learning and Development.

[45] Desmarais S., Curtis J. (1997). Gender differences in pay histories and views on pay entitlement among university students. Sex Roles.

[46] Davidson W.B., Beck H.P. (2006). Survey of academic orientations scores and persistence in college freshmen. J. Coll. Stud. Retent..

[47] Davidson W.B., Beck H.P., Milligan M. (2009). The college persistence questionnaire: Development and validation of an instrument that predicts student attrition. J. Coll. Stud. Dev..

[48] National Survey of Student Engagement (NSSE) (2017). NSSE 2017 U.S. Summary Means and Standard Deviations by Class and Sex.

[49] Bowler M. Dropouts Loom Large for Schools.

[50] Freire P. (1970). Pedagogy of the Oppressed.

[51] Wolters C.A., Hussain M. (2015). Investigating grit and its relations with college students’ self-regulated learning and academic achievement. Metacogn. Learn..

[52] Credé M., Tynan M.C., Harms P.D. (2017). Much ado about grit: A meta-analytic synthesis of the grit literature. J. Personal. Soc. Psychol..

[53] Ferry K. (2017). The Early Bird Gets the Best College Graduates? Korn Ferry survey Shows Best Time to Recruit Grads Is the Autumn of the Candidate’s Senior Year. https://www.kornferry.com/press/the-early-bird-gets-the-best-college-graduates-korn-ferry-survey-shows-best-time-to-recruit-grads-is-the-autumn-of-the-candidates-senior-year/.

[54] Brosnan S.F., De Waal F.B. (2003). Monkeys reject unequal pay. Nature.

[55] Krumrei-Mancuso E.J., Newton F.B., Kim E., Wilcox D. (2013). Psychosocial factors predicting first-year college student success. J. Coll. Stud. Dev..

[56] Taylor S.E., Scepansky J.A., Lounsbury J.W., Gibson L.W. (2010). Broad and narrow personality traits of women’s college students in relation to early departure from college. J. Coll. Stud. Retent..

[57] Tulgan B. (2016). Not Everyone Gets a Trophy: How to Manage the Millennials.

[58] Maddi S.R., Matthews M.D., Kelly D.R., Villarreal B., White M. (2012). The role of hardiness and grit in predicting performance and retention of USMA cadets. Mil. Psychol..

[59] Strayhorn T.L. (2013). What role does grit play in the academic success of black male collegians at predominantly white institutions?. J. Afr. Am. Stud..

[60] Van Koningsbruggen G.M., Hartmann T., Du J. (2017). Always on? Explicating impulsive influences on media use. Permanently Online, Permanently Connected: Living and Communicating in a POPC World.

[61] Bergman S.M., Fearrington M.E., Davenport S.W., Bergman J.Z. (2011). Millennials, narcissism, and social networking: What narcissists do on social networking sites and why. Personal. Individ. Differ..

[62] Passarelli A., Kolb D.A., Berg M.V., Paige R.M., Lou K.H. (2012). Using Experiential Learning Theory to Promote Student Learning and Development in Programs of Education Abroad. Student Learning Abroad: What Our Students Are Learning, What They’re Not, and What We Can Do about It.

